# How Does Tree Density Affect Water Loss of Peatlands? A Mesocosm Experiment

**DOI:** 10.1371/journal.pone.0091748

**Published:** 2014-03-14

**Authors:** Juul Limpens, Milena Holmgren, Cor M. J. Jacobs, Sjoerd E. A. T. M. Van der Zee, Edgar Karofeld, Frank Berendse

**Affiliations:** 1 Nature Conservation and Plant Ecology Group, Wageningen University, Wageningen, The Netherlands; 2 Resource Ecology Group, Wageningen University, Wageningen, The Netherlands; 3 Climate Change and Adaptive Land and Water Management, Alterra, Wageningen, The Netherlands; 4 Soil Physics and Land Management Group, Wageningen University, Wageningen, The Netherlands; 5 Institute of Ecology and Earth Sciences, University of Tartu, Tartu, Estonia; DOE Pacific Northwest National Laboratory, United States of America

## Abstract

Raised bogs have accumulated more atmospheric carbon than any other terrestrial ecosystem on Earth. Climate-induced expansion of trees and shrubs may turn these ecosystems from net carbon sinks into sources when associated with reduced water tables. Increasing water loss through tree evapotranspiration could potentially deepen water tables, thus stimulating peat decomposition and carbon release. Bridging the gap between modelling and field studies, we conducted a three-year mesocosm experiment subjecting natural bog vegetation to three birch tree densities, and studied the changes in subsurface temperature, water balance components, leaf area index and vegetation composition. We found the deepest water table in mesocosms with low tree density. Mesocosms with high tree density remained wettest (i.e. highest water tables) whereas the control treatment without trees had intermediate water tables. These differences are attributed mostly to differences in evapotranspiration. Although our mesocosm results cannot be directly scaled up to ecosystem level, the systematic effect of tree density suggests that as bogs become colonized by trees, the effect of trees on ecosystem water loss changes with time, with tree transpiration effects of drying becoming increasingly offset by shading effects during the later phases of tree encroachment. These density-dependent effects of trees on water loss have important implications for the structure and functioning of peatbogs.

## Introduction

Peatlands cover less than 3% of the Earth’s land surface but store almost 30% of all terrestrial soil carbon [1]. Raised bogs are open peatlands, dominated by *Sphagnum* mosses, with anoxic, acidic and nutrient poor conditions that hamper the establishment and growth of vascular plants, particularly trees [2]. Growing conditions for vascular plants could improve as the climate becomes drier and warmer. Climate change scenarios for the northern hemisphere indicate both an increase in average air temperature and more frequent drought events [3]. Drier and warmer conditions are known to improve vascular plant growth [4], [5], [6] as a result of reduced moss vitality [6], [7], as well as by increased availability of nutrients [8]. Indeed, recent woody plant encroachment in pristine [9], [10] and drained bogs [11], [12] has been attributed to warmer and/or drier conditions, as well as to changes in fire frequencies associated with a drier climate.

The effects of shrub and tree encroachment on peatland functioning and, ultimately, carbon sequestration are complex. Woody-plant dominated bogs could become net sources of atmospheric carbon if net photosynthetic rates and carbon fixation are lower than the decomposition rates of plant remains and accumulated carbon in the peat [12], [13]. The balance of these two processes will likely depend on the effects that trees have on their surrounding environment. Trees can potentially affect the bog environment through four main mechanisms: trees can dry soils by intercepting precipitation and transpiring water, they can increase soil nutrient availability by litter fall, they can reduce solar radiation by shading [14], [15], [16], and they can reduce wind influence by changing the aerodynamic properties of the bog surface [17]. These environmental changes could further facilitate the establishment and growth of vascular plants, potentially triggering positive feedbacks that could facilitate a shift towards a woody-dominated state [18], [19]. The evidence for the mechanisms described above is restricted, however, to correlative field measurements [14], [16], theoretical studies [15] and simulation models [20].

Whether trees affect the water balance towards drier conditions is one of the most important issues concerning tree encroachment in bogs. Studies on isolated trees suggest that the drying effect can be substantial [21], [22], but tree removal experiments [23] and modelling studies [24] have found inconsistent results. A major difficulty in assessing the effect of trees on the water balance is related to the different spatial scales at which trees affect the water cycle [25]. On one hand, trees can change the land surface albedo by absorbing solar radiation [26], [27] which can warm the air at large regional scales [25]. Trees also intercept precipitation and lose water by transpiration, which can also contribute to drier conditions in bogs. On the other hand, trees reduce the solar radiation and the humidity gradient under their canopy. The resulting cooler and moister microclimate can translate into less soil water evaporation and transpiration losses for plants growing under the tree canopy [28], [29]. The tree cooling effects on the understory may be particularly important for wet *Sphagnum* mosses since their lack of stomatal control leads to high evaporative water losses until the mosses dry out [17], [30], [31]. The interplay of the processes outlined above will determine the net effect of trees on the water balance.

We set out to test the effect of contrasting tree densities on the water balance of peat forming vegetation. Our working hypothesis was that trees would increase water loss from peat forming vegetation, and that the increase would differ between contrasting tree densities. To test this hypothesis, we conducted a three-year mesocosm experiment subjecting natural bog vegetation to three birch tree densities.

## Materials and Methods

### Experimental Design

We established a field experiment with mesocosms of bog vegetation planted with three birch densities (0, 1 and 2 birches per mesocosm) and five replicates per tree density using a randomised block design (Fig. S1). Each mesocosm was within a 9×10 m experimental plot planted with contrasting tree densities (0, 0.2 and 1 birch m^−2^) in order to minimise edge effects related to solar radiation, a major driver of physiological processes, and provider of energy used to evaporate water. The 9×10 m tree patches were not big enough to alter conditions in the atmospheric surface layer, although they did serve as wind-breakers to some extent.

The mesocosms (1.2 m diameter, 1.0 m deep, 20 cm above surrounding soil surface) were made from concrete rings with pond-foil at the inside, hydraulically isolating each mesocosm from its surroundings. The mesocosms were filled with 30–40 cm of natural bog vegetation above a layer of unfertilised milled *Sphagnum* peat (provenance Estonia). The layer of milled peat remained saturated with water throughout the experimental period, preventing differential effects on vertical transfer of water between the deeper and upper soil layers.

The experimental plots were arranged in 3 rows of 5 plots in a north-south direction in an experimental field with a short grass sward (Fig. S1). The experimental field was surrounded by low crops to the west, south and north allowing full exposure to the prevailing westerly winds. To the east, the field was partly bordered by taller trees, leading to slightly more sheltered conditions in blocks 1 and 2. There was a buffer of 1 meter between the plots at the south and north sides, but of 5 meters at the east and west sides (the two prevailing wind directions). Treatments were randomly assigned to the plots within each block. To minimise wind-break effects of one plot on the other, we constrained the spatial lay-out in such a way that no higher density plot bordered the windward (west) side of a lower density plot. The spatial lay-out did not interact with the tree density treatment effects, but did affect the water balance of the mesocosms for some seasons (Table 1). The experiment was conducted in Wageningen, The Netherlands between May 2007 and October 2010.

**Table 1:** Water balance components.

	Year	No Tree		Low Tree		High Tree		Statistics	
		*mean*	*SE*	*mean*	*SE*	*mean*	*SE*	*Treament*	*Block*
**Precipitation (P)**	2008	230^a^	2	236^a^	2	210^b^	5	F_df2_ = 11**	ns
	2009	187^a^	2	181^a^	3	143^b^	9	F_df2_ = 18***	ns
	2010	167^(a)^	2	155^(a)^	3	149^(b)^	9	F_df2_ = 3^(^*^)^	ns
**Irrigation (I)**	2008	29	–	29	–	29	–	–	–
	2009	33	–	33	–	33	–	–	–
	2010	71	–	71	–	71	–	–	–
**Runoff (R)**	2008	22	17	6	4	27	13	ns	F_df4_ = 4*
	2009	3^a^	3	4^a^	2	14^b^	3	F_df2_ = 9**	F_df4_ = 5*
	2010	0	–	0	–	0	–	–	–
**Storage (ΔW)**	2008	43	5	36	6	48	3	ns	ns
	2009	−59	6	−47	10	−39	8	ns	ns
	2010	−10^a^	2	−5^a^	6	11^b^	5	F_df2_ = 10**	F_df4_ = 3^(^*^)^
**Storage coefficient (S)**	2008&2010	0.3	0.02	0.3	0.01	0.3	0.01	ns	ns
**Evapotranspiration (ET)**	2008	2.0^ab^	0.2	2.3^a^	0.1	1.7^b^	0.1	F_df2_ = 7*	ns
	2009	2.8^a^	0.1	2.6^a^	0.1	2.1^b^	0.2	F_df2_ = 10**	ns
	2010	2.5^a^	<0.1	2.4^ab^	0.1	2.1^b^	0.1	F_df2_ = 8*	F_df4_ = 3^(^*^)^

Water balance components (means, SE, n = 5) were calculated over the summers of 2008–2010 (period V, calendar weeks 19–32). Water balance components (P, I, R and ΔW are in mm summer^−1^, S is in mm mm^−1^ and ET in mm day^−1^. Positive values of water storage refer to changes in water volume as a result of a net rise in the water table between the first and last date of period V, whereas negative values refer to changes in water volume as a result of a net decrease in the water table between the first and last date of period V. Different letters denote statistically significant differences between tree density treatments for the same year based on 2-way ANOVAs with treatment as factor and block as random factor. Ns = P<0.10, (*) = 0.10≥P<0.05, * = P≤0.05, ** = 0.05>P≤0.01, *** = P<0.01.

### Mesocosm Construction

Each mesocosm was equipped with a vertical drainage pipe (10 cm diameter) with an outlet at 10–15 cm below moss surface connected to an overflow container. The outlet prevented the water table from rising above the moss surface, and the overflow container ensured that all outflowing water could be measured. While the mesocosm set-up enabled careful quantification of the water balance, it constrained mechanisms that operate at larger spatial scales in natural bogs that stabilize the water table [32]. For example, the insulation between mesocosm and surroundings prevented lateral water supply from adjacent areas, such as pools. Consequently, faster water table draw down may occur in mesocosms than in natural raised bogs. To avoid severe desiccation of the peat during extended dry periods, we prevented water tables in the mesocosms to fall more than 50 cm below moss surface, corresponding to values observed in the field in e.g. Haaksbergerveen [33]. Here water tables generally did not fall below 30(40) cm under the moss surface for a range in tree densities (Table S1). When water tables in the mesocosms sporadically dropped below 50 cm under the moss surface (once in 2008 and 2009, twice in 2010), demineralized water was added to bring water tables back to 30–40 cm under the moss surface. Water was added through a hose hung within the vertical drainage pipe in small quantities at a time, thus avoiding direct rewetting of the moss surface. If water was added, the same volume was added to all treatments, maintaining the absolute differences in water tables between treatments. At the time of rewetting, water tables between treatments were not significant, making interference with the treatment effects unlikely.

### Plant Material

In April 2007 peat with natural vegetation was collected from a part of Soosaare bog in central Estonia (58° 33^,^ N, 25° 53^,^ E) authorized for commercial peat extraction, with permission from the private owner (Nick van de Griendt). The upper 30–40 cm of peat with vegetation was cut using a sharp knife, inserted into plastic boxes (40×50 cm, 30 cm deep), covered with plastic, and transported to Wageningen, The Netherlands. In Wageningen, the peat-vegetation samples were inserted into the mesocosms, using five boxes per mesocosm. The collected vegetation was characteristic of microsites with an intermediate water table (lawn or low hummock), with a species composition similar to Dutch peatlands [34]. The vegetation was dominated by *Sphagnum magellanicum* with some *Sphagnum fuscum and Sphagnum rubellum*. The herb cover varied between 10 and 20%, mainly consisting of the evergreen dwarf shrubs *Calluna vulgaris*, *Andromeda polifolia* and *Vaccinium oxycoccus* and the graminoid *Eriophorum vaginatum*.

### Planting

The birch (*Betula pendula*) saplings planted inside the mesocosms were taken from the Bargerveen, a bog reserve in the Netherlands (52°42′N, 7°03′E) with permission from the owner (the Dutch Forest Service). In October 2007, birch saplings of 1.5–1.8 m tall were carefully extracted from the peat, minimising root damage as much as possible. On the same day, all saplings were planted in the mesocosms. For the birch planting, we opened the moss vegetation with a sharp spade, inserted the roots into the peat matrix above the water table, and pressed back the parted vegetation. To keep the disturbance of the peat vegetation equal across treatments, we also parted, and pressed back, the moss vegetation in the treeless mesocosms. None of the transplanted birch saplings died, although the top of one birch sapling in a low-tree density mesocosm was damaged to such an extent that the sapling resprouted from side-buds. The water loss for this mesocosm remained in the lower range of its treatment. The birches (*Betula pendula*, some *Betula pubescens*) planted outside the mesocosms in the plots were 1.5 m tall on average, bought from a commercial source, and were planted early in May 2007.

Our choice for birch, the most common tree species in West and Central European bogs, allowed us to compare treatment effects during a vegetated and non-vegetated season. The birches used for the mesocosms had a superficial (10–20 cm below moss surface) rooting pattern, characteristic of trees growing in bogs. The planting densities (0.2–1 birch m^−2^) and sapling height (1.5–1.8 m) in our experiment were based on the range that can be found in Dutch bogs. In comparison: birch tree stands in Haaksbergerveen (52°7′N, 6°46′E), another bog reserve in the Netherlands, range in tree density from 0.2 to 3.6 trees m^−2^ of small trees (0.6–4.1 m height and 0.8–8.0 cm diameter), with a maximum tree age of 32 years [33].

### Maintenance Experiment

In 2009 and 2010 the trees outside the mesocosms were thinned to prevent their canopy from becoming denser than that of the trees within the mesocosms. To avoid fertilisation of the mesocosms by the nutrient richer leaves of the birches planted outside the mesocosms, we replaced all birch leaves blown into the mesocosms by an equal dry weight of birch leaves from the Haaksbergerveen, each autumn. In the course of 2008 the graminoid *Eriophorum vaginatum* expanded strongly in all mesocosms, irrespective of treatment, presumably because this deep-rooting species accessed the nutrients in the deeper milled peat layer. To avoid outshading of the *Sphagnum* moss, we clipped and removed all *Eriophorum* shoots from the mesocosms in the winters of 2008–2009 and 2009–2010. The clipping treatment resulted in less vigorous *Eriophorum* growth, but did not increase mortality of this species.

### Measurements

#### Water balance mesocosms

Precipitation, runoff, and water table level were measured every Monday morning at weekly intervals between September 2007 and October 2008. For 2009 and 2010, we only measured at weekly intervals between May and September, as the treatments only started to differ in the course of the growing season. Evapotranspiration (ET, in mm day^−1^) from the mesocosms was estimated from the observations as:
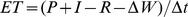
where P is precipitation in plots without trees, and throughfall (precipitation minus canopy interception) in plots with trees, I = irrigation, R = runoff, ΔW = change in water storage due to fluctuations in water table level, Δt = measurement period of P, I, R and ΔW, in days.

Precipitation (P) was measured in mm using common-garden rain gauges (Nortene, pluvius 2), one in each plot, placed directly to the west of each mesocosm. The opening was kept at 30 cm above the ground, level with the surface. Consequently, we measured precipitation in plots without trees, but throughfall in plots with trees. Our choice for one rain gauge per plot necessitated the use of an inexpensive system instead of a more advanced tipping bucket system. We compared daily sums of the rain gauges with those of a tipping bucket system placed about 200 m distance from the experimental field between 12 June and 28 August 2009. The precipitation measured in the control (R^2^ = 0.86, slope 0.97; linear regression) and low density plots (R^2^ = 0.87, slope 0.92) closely followed those measured with the tipping bucket system, supporting the accuracy of the gauges. The precipitation in the high density treatment (R^2^ = 0.80, slope 0.66) differed somewhat more, however, indicative of the uneven passage of precipitation through a canopy. As measurements of precipitation under a canopy are sensitive to the position of the rain gauges, they may lead to errors in evapotranspiration estimates. To explore the potential importance of rain gauge position on our evapotranspiration estimates, we explored the variability in precipitation among the replicates within each treatment. To this end we calculated the coefficient of variation (CV) using data on daily precipitation sums between 12 June and 28 August 2009. The CV decreased exponentially with the daily sum for all treatments. Over the entire period of 77 days, the CV was 0.6, 0.9 and 2.1% of the total precipitation sum for the control, low density and high density treatments respectively, suggesting the influence of rain gauge position on our evapotranspiration estimates was negligible.

Irrigation (I) equalled the water sporadically added to mesocosms to avoid severe desiccation of the peat. Water was added below the moss surface once in 2008 & 2009, and twice in 2010. See section entitled *mesocosm construction* for more details.

Runoff (R) was determined by measuring the volume of water in the overflow containers. Runoff was expressed in mm by dividing the volume (L) by the area of the mesocosm (m^2^).

Water storage change (ΔW) was calculated as:
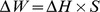
where ΔH is the change in water table level (mm) and S the storage coefficient (mm/mm).

Please note that this approach does not include changes in the volume of retained water above the water table. At a weekly scale this means a slight underestimation of ET during weeks in which the moss layer above the water table dried out and a slight overestimation during weeks in which moisture in the moss layer was replenished again by precipitation. At the seasonal scale we adopted, these weekly fluctuations and possible errors are mostly eliminated. Consequently, the potential error in the water balance is restricted to differences in the amount of water retained above the water table between the first and last date of the time series over which ET was averaged (see section entitled *evapotranspiration*), making this error a negligible (<5%) component of the seasonal water balance.

The storage coefficient, S, is the volume of water per unit of surface area per unit of water table change. The storage coefficient of the upper 30–40 cm of peat was measured for each mesocosm both in the first (2008) and final year (2010) of the experiment by alternately pumping out water of the mesocosms and measuring the resulting change in water table. The 2008 and 2010 values were averaged, yielding mesocosm –specific values of S that were used to calculate mesocosm-specific changes in water storage (ΔW, see previous section). S was higher in 2010 than in 2008, but remained unaffected by depth or treatment for both measurement times. We refer to Table S2 for mesocosm-specific values of S and more detailed information on measurement procedures.

#### Evapotranspiration

We calculated the weekly mesocosm evapotranspiration and averaged it over five periods (I–V). The periods were based on potential evapotranspiration (ETp) to account for seasonal differences in solar radiation and vegetation development. The period lengths ranged from 4 to 22 weeks (Table 2), and differed 0.8 mm day^−1^ in ETp on average. Periods I and II approximately cover those weeks where trees had no, or only few leaves, whereas periods III–V represent those weeks where trees were in leaf. Period V ran from late spring to late summer (weeks 19–32), covering the time of the year with most incoming solar energy and best developed canopies. To facilitate data interpretation we refer to period V as summer. The potential evapotranspiration ETp was computed according to the method proposed by Hargreaves and Samani [35], based on daily air temperature. We used daily temperature records from the nearby meteorological station “Haarweg” (Wageningen, Netherlands). We used ETp for data presentation and interpretation only; it does not affect the relative differences among treatments.

**Table 2:** (Time) periods and their average climate.

Period	Length	Leaves	Sun	T_min_	T_max_	Vp	Wind	Dates	Calender weeks	
	weeks	yes/no	h day^−1^	°C	°C	kPa	m s^−1^		2007	2008
I	22	no	3	1	10	0.9	3	15 Oct–16 Mar	42–52	1–11
II	7	no	4	3	16	1.0	2	17 Mar–6 Apr & 24 Sep–14 Oct	39–41	12–14
III	6	yes	5	5	21	1.3	2	7–20 Apr & 25 Aug–20 Sep	35–38	15–16
IV	4	yes	6	7	23	1.3	3	21 Apr–5 May & 11–24 Aug		17–18 & 33–34
V	13	yes	7	9	26	1.5	3	6 May–10 Aug		19–32

Main climatic variables have been derived from the closest meteorological station: de Haarweg, Wageningen. Climatic variables were measured at 1.5 m above soil surface and represent weekly averages. Length = number of weeks within a period, Leaves = presence of leaves on the birch trees, Sun = average number of sun hours per week, Vp = Vapour pressure.

#### Vegetation

As vegetation composition and structure may affect evapotranspiration, we monitored cover of the understory and birch canopy. Cover was monitored per species in permanent quadrants, using the point intercept method [36] each summer in August-September between 2008 and 2010. This entailed fixing a frame of 25×37.5 cm and with a 2.5 cm grid above this quadrant. At 150 points a needle could be lowered to the moss surface. We recorded each species that was touched by the point of the needle, distinguishing between living and dead plant material. Number of touches per species were later aggregated into three categories: vascular plants, litter, and moss.

Cover of the tree canopy was measured as the leaf area index (LAI, the one-sided leaf surface area per unit of soil area) at about 50 cm height. For 2009 and 2010 LAI was measured using an LAI-2000 (LI-COR) on an overcast day. In 2008 the LAI was derived from measurements of basal area (Table S3). The LAI was based on 12 measurements under the birch canopy and 4 outside the birch canopy at each plot corner. The measurements were taken along a cross through the centre of each plot, with one measurement on each of the 4 compass points of the mesocosms and the other 8 at regular intervals between the mesocosm and the plot edge.

#### Subsurface temperature

To explore the effect of the birch saplings on the temperature under the canopy, peat surface temperature was measured in two mesocosms per treatment over July 2010. We inserted a thermistor (T 107, Campbell Scientific, UK) enclosed in a tightly fitting plastic zip-lock bag 1–2 cm below the moss surface in the middle the mesocosms. After insertion the moss carpet was pressed back to minimise differential heating of bag and thermistor. Measurement intervals were set at 5 minutes. One of the two thermistors in the low density treatments malfunctioned, leaving us with one replicate for surface temperature in the low density treatment.

### Statistical Analyses

After controlling for statistical assumptions, we tested if evapotranspiration and the water balance components differed significantly between the birch treatments, using a 2-way ANOVA, with birch density as fixed factor and block as random factor for each period separately. Precipitation sums were square root transformed prior to statistical analysis. Treatment effects on the abundances of vascular plants, litter and moss were analysed with a repeated measure ANOVA, using year as the between subject factor and birch density as the within subject factor. The relationships between temperature and LAI were analysed with linear regression. All statistical analyses were performed with SPSS (version 19) for Windows.

## Results

### Tree Density

**Figure 1:**
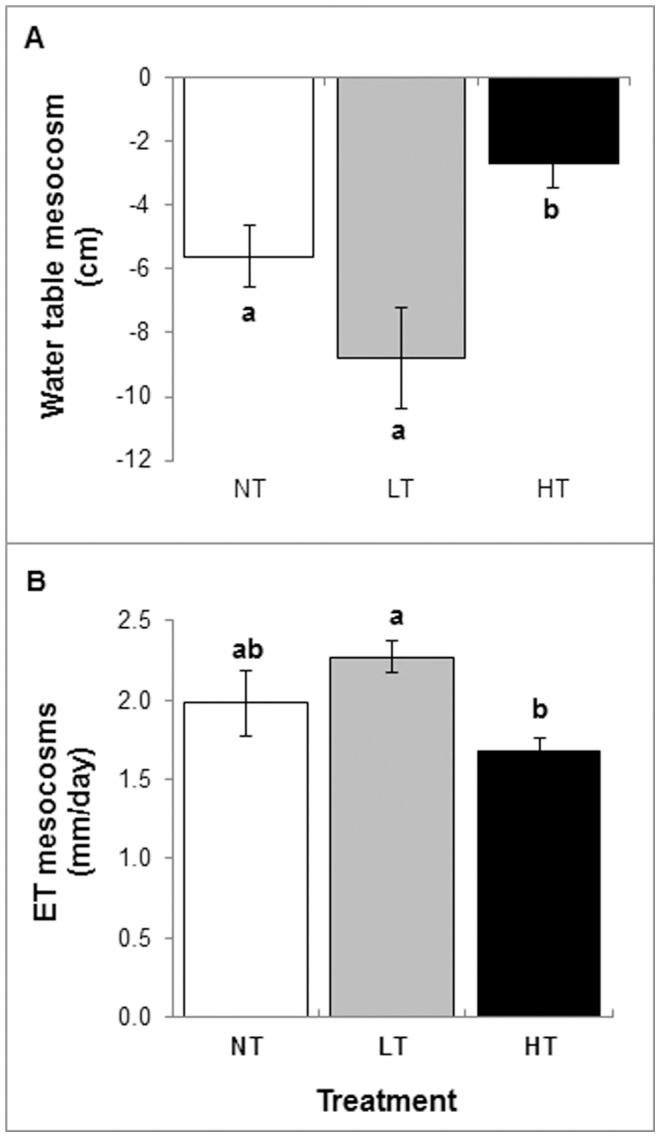
Effects of tree density on water table (panel A) and mesocosm evapotranspiration (panel B). Bars represent means +1 SE (n = 5) per birch density treatment. NT = control without trees, LT = low tree density, HT = high tree density. Mesocosm evapotranspiration was averaged over the first summer (period V, calendar week 19–32, 2008). Water table values refer to the situation at the end of period V, as measured at the beginning of week 33. Water table level was measured relative to a fixed point (the overflow outlet), which was 10–15 cm below the moss surface. Different letters above the bars denote statistically significant (*P*<0.05) differences between tree density treatments based on 2-way ANOVAs with treatment as factor and block as random factor.

Over the first summer (period V, 2008), tree density affected mesocosm water loss leading to significant differences in the water table between experimental treatments (Fig. 1A). The deepest water table was found in mesocosms with low tree density (LT), whereas mesocosms with high tree density (HT) remained wettest with the highest water tables. The differences in water table mainly resulted from differences in evapotranspiration (Fig. 1B). Mesocosm evapotranspiration was consistently highest in the low tree density (LT) treatment and smallest in the high tree density (HT) treatment, with mesocosms with no trees (NT) being in an intermediate position, suggesting a non-linear effect of tree density on evapotranspiration. The following two experimental years confirmed the effect of high tree density on mesocosm water table (Table 1), despite differences in summer precipitation between years. Tree density affected the amount of precipitation passing the canopy (throughfall) and water flowing out of the mesocosms (run-off; Table 1). In the LT treatment, throughfall did not differ from precipitation in the NT treatment, showing that the LT tree canopy remained too open to intercept much precipitation (Table 1). In the HT treatment, however, the tree canopy intercepted about 10% of the precipitation. Run-off peaked in the HT treatment. The highest run-off was measured in the most sheltered plots, notably the HT plots in blocks 1 and 2 (for position blocks see Fig. S1).

Tree canopy reduced temperature fluctuations: the average temperature just below soil surface was 1–2 degrees lower in the mesocosms with trees than for mesocosms without trees (data not shown). This tree effect was mainly due to lower maximum temperatures, consistent with lower radiation at the soil surface under the tree canopy, although we cannot exclude that small differences in surface moisture may have contributed to this effect. The maximum temperature was on average 5 degrees lower in the HT treatment and 2 degrees lower in the LT compared to the NT treatment (Fig. S2). The minimum temperature at soil surface remained unaffected by tree density (data not shown).

### Seasonality

**Figure 2:**
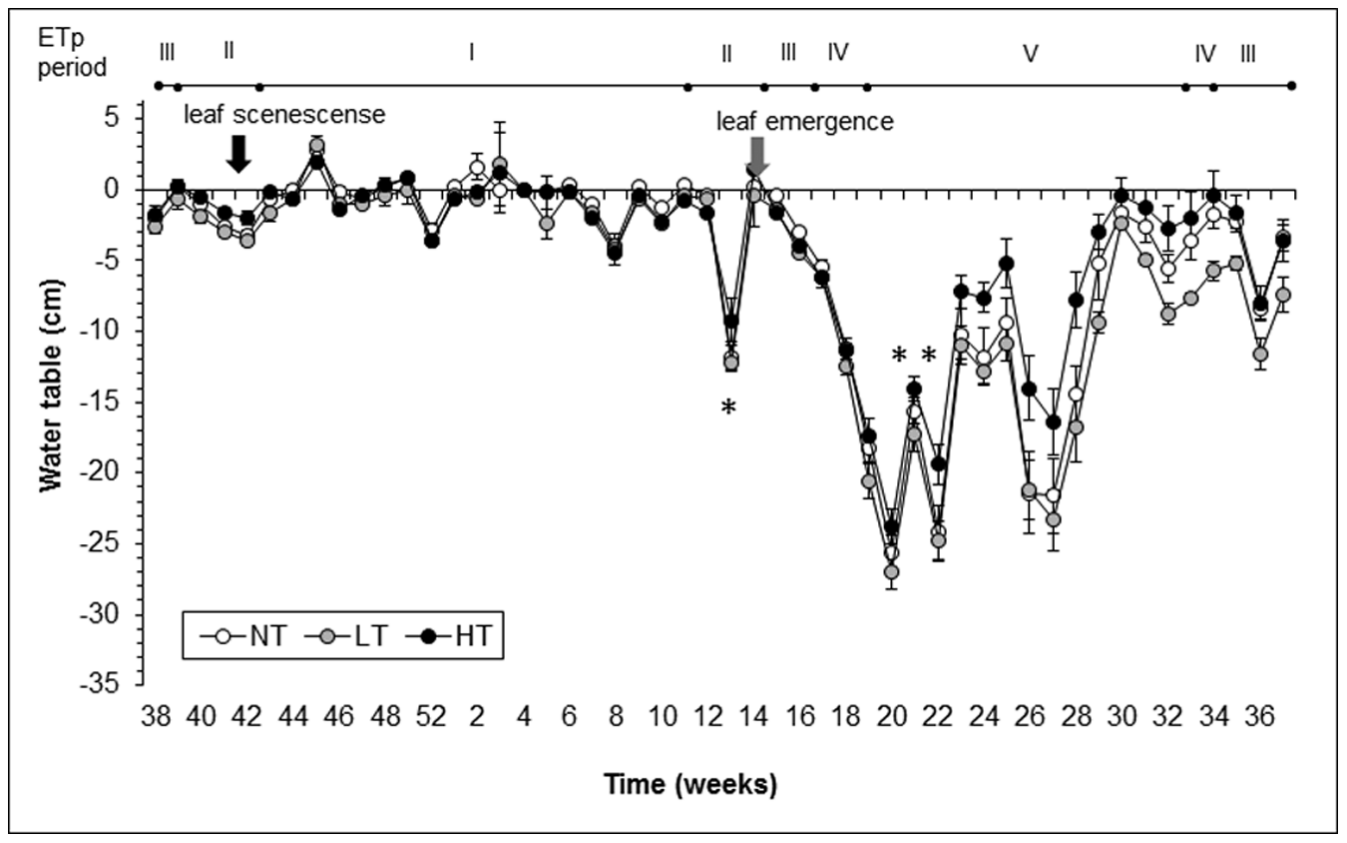
Seasonal changes in tree density effects on mesocosm water table. Bars represent mean water tables ±1 SE (n = 5) in cm relative to a fixed point per week, from week 38 in 2007 (38) until week 37 in 2008 (37). Positive values indicate a water table closer to the surface. Water table level was measured relative to a fixed point (the overflow outlet), which was 10–15 cm below the moss surface. Birch density treatments are identified by differently shaded bullets. NT = control without trees, LT = low tree density, HT = high tree density. Arrows indicate onsets of leaf senescence in 2007 and leaf emergence in 2008. * = week during which storage coefficient has been determined, ** week in which demineralized water has been added to each mesocosm. ETp periods indicates periods (I-V) differing in solar radiation and potential evapotranspiration, over which evapotranspiration has been averaged for Figures 1, 3 and 4. Note: mesocosm trees were planted in December 2007.

**Figure 3:**
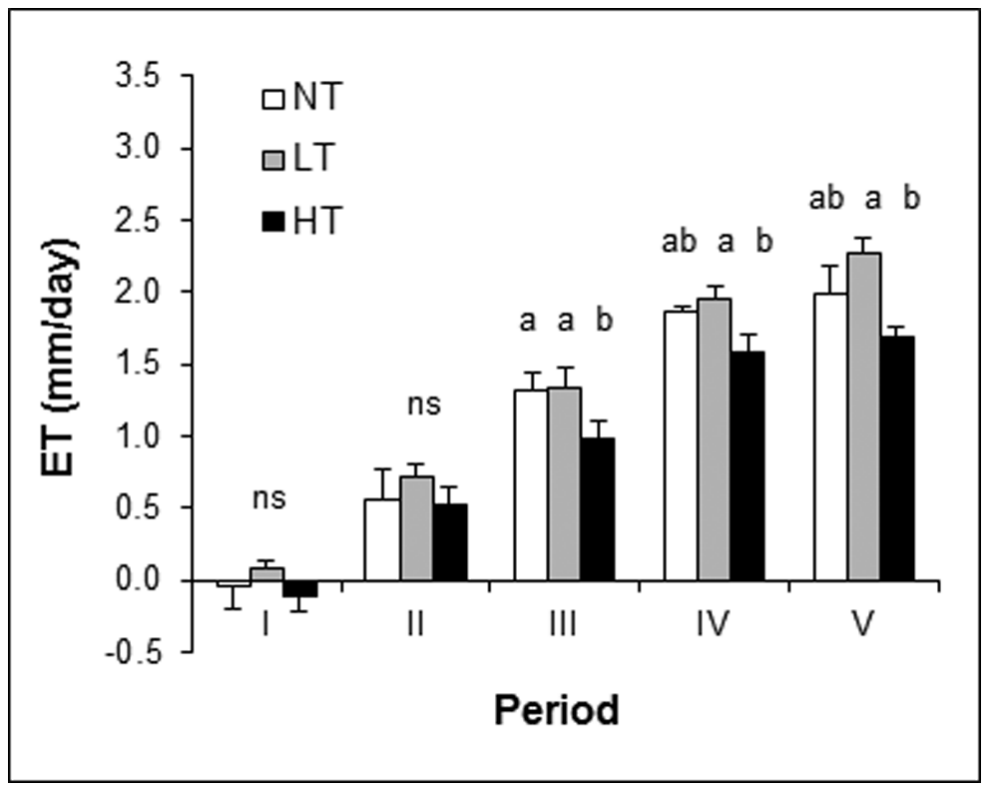
Seasonal changes in tree density effects on mesocosm evapotranspiration. Bars represent means +1 SE (n = 5) per birch density treatment averaged over periods (I–V) in order of increasing atmospheric demand for water. Periods I and II cover late autumn -early spring, whereas periods III–V represent mid spring-mid autumn (Table 2). Measurements spanned 1 year from week 38 in 2007 until week 37 in 2008, the only year for which we had water table data for all seasons. NT = control without trees, LT = low tree density, HT = high tree density. Different letters above the bars denote statistically significant (*P*<0.05) differences between tree density treatments within a period based on five separate 2-way ANOVAs with treatment as factor and block as random factor, one ANOVA for each period.

Trees affected the water table from early spring (April 2008, week 16) onwards, which was after leaf emergence had taken place (Fig. 2). In late summer (September 2008, week 36) the tree effect on the water table ceased as leaves were shed and precipitation increased. These patterns were mirrored by mesocosm evapotranspiration (Fig. 3). Trees affected mesocosm evapotranspiration between spring and summer (periods III–V) especially in the high tree density (HT) treatment.

Besides tree density and season, the spatial arrangement of the plots also affected mesocosm evapotranspiration, resulting in significant block effects for periods I-III (data not shown). The lowest evapotranspiration, and smallest difference between tree density treatments, was found for the most sheltered blocks 1 and 2 (Fig. S1). This block effect on evapotranspiration was no longer significant in late spring (period IV) and summer (period V, Table 1).

### Vegetation

**Figure 4:**
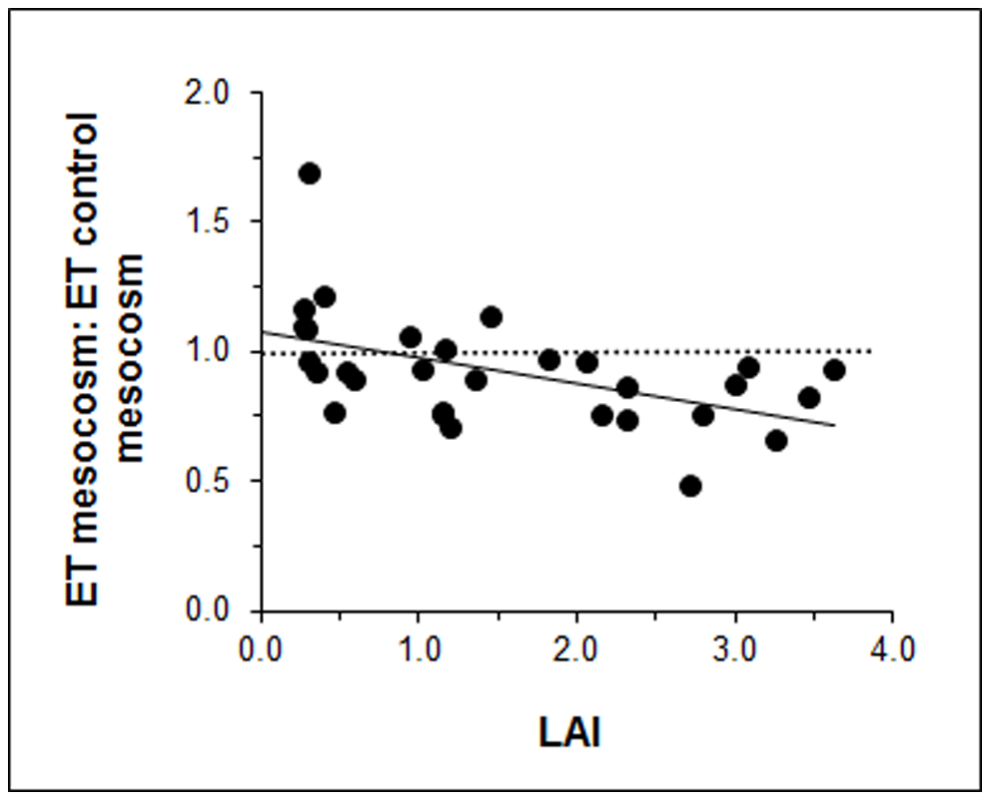
Relationship between plot-LAI and mesocosm evapotranspiration (ET) for the summers of 2008, 2009 and 2010. ET of the mesocosms with trees (LT and HT) were averaged over the summer (period V) for each year separately and standardized by dividing by the ET from mesocosms without trees (NT mesocosms). Symbols above the dashed line indicate a higher evapotranspiration than NT mesocosms, whereas symbols below this line indicate lower evapotranspiration than NT mesocosms. The solid line indicates a weak, but significant, (*P*<0.05), linear relationship (linear regression, R^2^ = 0.25, y = 0.1x+1.1).

Tree growth resulted in a denser and broader tree canopy, as reflected by the Leaf Area Index (LAI). For the low density treatment, LAI increased from 0.3 in 2008 to 1.5 in 2010, whereas it increased from 1.2 to 3.3 for the HT treatment. Relating summer evapotranspiration to LAI, revealed a significant, albeit weak (R^2^ = 0.34; linear regression), negative relationship (Fig. 4). Most of the mesocosms in which evapotranspiration exceeded that of the NT treatment had an LAI below 1, although variability was considerable. Above LAI = 2, mesocosms with trees had a consistently lower evapotranspiration than mesocosms without trees. Trees inside the mesocosms grew at a slower rate than those outside the mesocosms, presumably because of shallower rooting depth (maximally up to 30(40) cm below surface) and lower nutrient availability. Consequently tree LAI inside and outside the mesocosm diverged over the years (Table S3), despite pruning. In 2008, mesocosm-LAI did not differ from plot-LAI for all treatments. In 2009, mesocosm-LAI was lower than plot-LAI for the HT treatment only, whereas for 2010, mesocosm-LAI was about half that of plot-LAI in both the LT and HT treatments.

In the understory, vascular plant abundance increased steeply in the course of 2008 and 2009, irrespective of tree density (Table 3). The increase in vascular plants was mainly attributable to *Eriophorum vaginatum*. This deep rooting graminoid species changed from having a sparse cover with a few inflorescences to forming dense tussocks with many inflorescences. Presumably, its roots reached the (unfertilised) milled peat underlying the 30–40 cm of natural bog vegetation, giving access to the nutrients released by this layer (see methods). Alternatively, the step increase in N deposition from <0.4 g m^2^ yr^−1^ in Estonia to close to 4.0 g m^2^ yr^−1^ in Wageningen, may have stimulated *Eriophorum* production [37]. Over 2010, vascular plant abundance stabilised in the NT and LT treatments, but decreased in the HT treatment, leading to a significant year × treatment interaction. In the HT treatment, *E. vaginatum* had a growth form characteristic of shady habitats, with longer, thinner leaves and fewer inflorescences than the other treatments. The decrease in vascular plant abundance in the HT treatment was accompanied by a response in in vascular plant litter. Between 2009 and 2010 litter decreased for the NT and LT treatments, but remained stable for the HT treatment. The deviating pattern for the HT treatment suggests light levels under the tree canopy became suboptimal for understory vascular plants, resulting in higher *Eriophorum* mortality in this treatment in 2010. Moss cover declined sharply over the years irrespective of tree density, although the decline seemed sharpest in the HT treatment (Table 3). The growth form of the mosses in the HT mesocosms was characteristic of shady habitats with slightly smaller capitula, longer stems and less frequent side branches, resulting in an overall looser carpet structure.

**Table 3:** Vegetation composition understory.

	Year	No Tree		Low Tree		High Tree		Statistics		
		*mean*	*SE*	*Mean*	*SE*	*mean*	*SE*	*Treatm*	*Year*	Treatm*year
**Vascular plants**	2007	28	3	18	3	31	5	ns	F_ε1.0,df2_ = 159***	F_ε1.0,df4_ = 9***
(% frame)	2009	118	10	122	15	160	49			
	2010	167	20	170	18	44	21			
**Litter**	2007	6	1	9	2	12	3	F_df2_ = 4^(^*^)^	F_ε1.0,df2_ = 29***	F_ε1.0,df4_ = 7**
(% frame)	2009	185	16	142	12	132	13			
	2010	97	12	65	5	140	9			
**Moss**	2007	95	1	92	2	95	2	ns	F_ε0.9,df1.8_ = 82***	F_ε0.9,df3.6_ = 3^(^*^)^
(% frame)	2009	66	9	65	5	69	1			
	2010	49	9	45	6	28	7			

Abundances (means, SE, n = 5) of vascular plants, litter and moss in the mesocosms over the experiment (2007–2010). Data on 2008 are missing, due to a malfunctioning voice recorder. 100% of frame = species hit in all 150 points of the point-quadrat frame (see methods). For plants with horizontal (planar) leaf orientation 100% frame roughly corresponds to LAI = 1. Values over 100 indicate multiple hits per point. Statistics give results of repeated measures ANOVAs with treatment as within subject factor and year as between subject factor. Ns = P<0.10, (*) = 0.10≥P<0.05, ** = 0.05>P≤0.01, *** = P<0.01.

## Discussion

### Effects of Trees on Water Loss

We found that trees increased mesocosm evapotranspiration only at a leaf area index (LAI) well below 1, whereas at LAI = 2 or higher, trees reduced evapotranspiration relative to treeless mesocosms (Fig. 4). These results contrast sharply with the generalizations predicting a net linear negative drying effect of trees on their environment [14], [21]. Earlier studies showing strong drying effect of trees [21] focussed on isolated (birch) trees, neglecting density-dependent effects. In contrast, a recent modelling study by Kettridge et al. [24] suggested neutral effects of tree density (spruce) on ecosystem evapotranspiration up until (very) high tree densities. Interestingly, our results suggest that tree density strongly determines the net effect of trees on the total water balance of bogs, pointing towards a non-linear relationship between water balance and tree density. Our results are consistent with Strilesky and Humphreys [38] who measured lower evapotranspiration for bog vegetation with (spruce) trees compared to treeless vegetation within the same bog. Central to the tree effect seems the degree in which tree transpiration is offset by reduced evapotranspiration of the shaded understory, making the net outcome sensitive to tree growth, canopy structure, species composition of the understory and water table.

### Shading Effects on Understory Evapotranspiration

We explored if, after accounting for reduction in understory evapotranspiration by shading, the resulting trends in mesocosm evapotranspiration rates would be physically plausible. To this end, we used a well-known, simple theoretical framework that describes evapotranspiration for well-watered, radiation-limited conditions. Under these conditions, and for the timescale considered in our study, evapotranspiration is approximately proportional to the energy available at the surface [39]. For the Dutch climate it has been shown that the evapotranspiration can then be estimated from the solar radiation and the air temperature alone [40]. This avoids the need to know the humidity gradient between the surface and a reference level in the atmosphere [41], [42]. Thus, for approximately similar average air temperature, differences in evapotranspiration of a well-watered system can then be evaluated from differences in the solar radiation reaching the surface. As the tree copses were fairly small, enabling easy mixing with the air outside the canopies, it seems reasonable that average air temperature was comparable between the treatments, meeting the first assumption above. It also seems reasonable to assume that mesocosm evapotranspiration was limited by radiation at the seasonal scale, as mesocosm evapotranspiration increased with solar radiation, i.e. it gradually increased from winter (period I) to summer (period V) for all treatments (Fig. 3).

Using this theoretical framework, we estimated how much the tree canopy could reduce understory evapotranspiration by absorbing solar radiation. We assumed that (i) understory evapotranspiration was proportional to solar radiation (see above, and the cited literature); (ii) the birch canopy was dense and homogenous (LAI = 3, the maximum LAI observed in our study); (iii) the birch canopy absorbed solar radiation according to Lambert-Beer’s law:


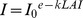
, where I is the radiation below the tree canopy, I_0_ is the radiation above the tree canopy, and k is a species-specific light extinction coefficient [43]. For the light absorption coefficient k of the birch canopy we assumed the birch-specific value of 0.57 [44]. In that case and for LAI = 3 it follows from Lambert-Beer’s law that such a tree canopy could reduce radiation below its canopy to about 20% of the value at the top of its canopy. This implies that understory evapotranspiration would be likewise reduced to, roughly, 20% relative to a treeless condition (the NT mesocosm).

We expressed the contribution of tree transpiration to mesocosm evapotranspiration relative to that of the treeless (NT) mesocosms. Evapotranspiration in the HT mesocosms was 80% of that in the NT mesocosms (Fig. 1B). Of this 80% about 20% could be attributed to the shaded understory (estimated above). Thus, according to our rough estimate, the contribution of tree transpiration to mesocosm evapotranspiration would amount to ∼60% of the evapotranspiration in the NT mesocosms. Assuming evapotranspiration from the bog vegetation in the NT mesocosms approached the international standard reference transpiration of well-watered, unstressed grassland [45], tree transpiration in our experiment amounted to 60% of the standard reference transpiration. Although comparable to values measured for mixed forests in the Netherlands [46], [47], 60% is relatively low, implying suboptimal growing conditions corresponding to the slow tree growth in water logged peatbogs [48].

Of course the above calculations are a gross simplification of all processes and state-variables involved, but they do suggest that trees may reduce water losses if these water losses for the treeless state are relatively high to begin with, and trees grow slowly, as is both the case for the moist-wet lawn conditions simulated in our experiment. These observations are consistent with work by Lafleur [49] who found that increases in vegetation cover reduced evapotranspiration of a wet sedge site by comparing evapotranspiration before and after leaf emergence. How sensitive our treatment effects are to changes in surface moisture condition cannot be quantified with our approach. However, since our treatment effects were consistent for three years (Table 1) despite differences in sun hours and precipitation (Table 2), it seems this sensitivity is limited at the seasonal scale adopted in our study. In drier (continental) climates, where the environment is more favourable for tree growth and rewetting of the surface moss-layer by precipitation occurs less frequently than in the temperate (Dutch) climate, the drying effects of trees may be stronger than observed in our study.

### Potential Role of Experimental Artefacts

Mesocosm experiments are a valuable bridge to modelling and field observation studies, as they allow a higher degree of control in the manipulation of conditions [50]. Our mesocosms separated the effect of trees from that of their environmental setting. This control comes of course at a cost: not all feedbacks that exist in the field can be reproduced perfectly. Below, we address three differences between our experimental setting and that of field conditions, and explore if and how they could have influenced our results.

Firstly, in our experiment, the mesocosms were hydraulically isolated from their surroundings, preventing the stabilizing influence of lateral water recharge from wetter adjacent areas that would normally occur in bogs. To prevent unnatural desiccation we constrained water table draw down to values measured in a natural Dutch bog (Table S1) by sporadically adding water. Adding water could have biased the results by rewetting the moss surface, prolonging moss evaporation in the mesocosms beyond that observed in the field. Although water additions may have affected overall water loss from the mesocosms by stimulating upward capillary transport, interference with treatment effects seem unlikely as we only added water once a year in 2008 & 2009 and twice in 2010, keeping well below (30 cm) the moss surface at a time that water tables did not differ significantly between the treatments (Fig. 2).

Secondly, the mesocosms were positioned in a field with a short grass sward instead of in a peatland, creating a potential contrast in surface moisture between mesocosm and surroundings. Consequently, oasis effects could have enhanced the water losses from the mesocosms above those normally reported for bog vegetation. However, when we compare the summer ET losses from our mesocosms with those reported for other raised bogs [51], [52] our mesocosm values (1.7–2.8 mm day^−1^) fall in the lower, not the higher, range of estimates for bogs (2.2–3.3 mm day^−1^), suggesting oasis effects were negligible.

Finally, to minimise edge effects on solar radiation, we planted trees outside the LT and HT mesocosms (see methods). The trees outside the mesocosms grew faster than the trees inside the mesocosms, resulting in a discrepancy in LAI between trees surrounding the mesocosms and those planted in the mesocosms by 2010. Whereas we cannot eliminate that this discrepancy influenced the 2009 and 2010 results, it does not explain why we found the most pronounced tree effects for 2008, when both mesocosm-LAI and plot LAI were in agreement.

In conclusion, we think that despite its potential shortcomings, our approach is technically sound to assess the effects of tree density on water losses of peat vegetation at a relevant scale. Every scientific approach has its own advantages and disadvantages. Although field monitoring and modelling have been more commonly used in peatland ecology, hydrology and micrometeorology [9], [17], [24], we strongly believe that combining their valuable results with experimental data, like that presented here, is a necessary step to further improve our understanding on the dynamics of these ecosystems.

### Consequences for Tree Encroachment and Bog Functioning

Obviously, our mesocosm results cannot be directly scaled up to natural peatbogs with a complex arrangement of dry and wet microsites and different aerodynamic surface properties. However, the systematic tree density effects in our experiment imply that as bogs become colonized by trees, the effect on the water balance may change in time and space. The initial drying effect caused by isolated trees [21] decreases as tree stands become denser, indicating that sparse tree colonisation from bog edges [53] could facilitate further tree invasion while continued tree growth in place would lead to a negative feedback on water loss. As the feedback of trees on the water cycle weakens (Fig. 1), other feedback mechanisms mediated through increased shading such as changes in understory species growth form, cover (Table 3) and composition [30], reduced wind speed [29], or faster nutrient cycling [16] become increasingly relevant to explain the effects of trees on the functioning of bog ecosystems Our results also suggest that the spatial configuration of trees may be an important feature to understand their effects on the water balance in bogs. If our inferences are correct, clustered trees will likely have less drying effect than single trees. Since recruitment of trees occurs mainly on moist to dry microsites (lawns and hummocks) within a bog [53], the spatial configuration of these microsites will co-determine the effect that trees have on the water balance. As such, colonization of wide-spread smaller microsites will have larger effects on the water balance than colonization of an equally large area of more clustered patches.

A non-linear effect of tree density on the water balance may have large implications for patterns of tree recruitment in bogs and our understanding of interspecific interactions in these ecosystems. Our results suggest that isolated trees, or patches with low tree density, are more effective in drying their surroundings, and therefore may facilitate the establishment of other woody species in bogs more strongly than high tree density patches [4], [5], [6]. The non-linear effect of trees on the water balance of bogs could translate into non-linear effects on plant recruitment, particularly when associated with deeper water tables. Non-linear effects of shade on plant water relations and plant facilitation have been found in several terrestrial ecosystems [54], [55]. Although plant facilitation has been poorly studied in peatlands [56] compared to other types of terrestrial ecosystems [57], our results suggest that the effect of trees on their environment is important.

### Conclusions

We conclude that as peatlands become colonized by trees, the effect of trees on ecosystem water loss changes in time and space. The initial drying effect of single trees on peatlands decreases as the denser canopy starts shading the moss surface. Non-linear effects of tree density on water loss may have large implications for patterns of tree recruitment in peatlands and our understanding of the feedbacks between vegetation composition and water balance.

## Supporting Information

Figure S1 **Layout experimental field with position plots and blocks.**
(DOCX)

Figure S2 **Tree density effects on subsurface temperature.**
(DOCX)

Table S1 **Water table fluctuations under natural field conditions.**
(DOCX)

Table S2 **Storage coefficients (S) of mesocosms: values and measurement procedure.**
(DOCX)

Table S3 **Leaf area index (LAI) of plots and mesocosms.**
(DOCX)
