# Supplementary material for: How Does Tree Density Affect Water Loss of Peatlands? A Mesocosm Experiment
Source: PLoS One. 2014 Mar 14;9(3):e91748. doi: 10.1371/journal.pone.0091748 (PMC3954773; doi:10.1371/journal.pone.0091748)
Supplement: Figure S1 — Layout experimental field with position plots and blocks. (DOCX) [file pone.0091748.s001.docx]

N

1

1

1

2

2

2

4

5

3

3

3

4

4

5

5


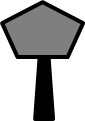


**Figure S1.** **Layout experimental field with position plots and blocks.** Squares indicate plots, numbers within squares correspond to blocks. Mesocosms were positioned in the centre of the plots. Dominant wind direction was from the west (left). Block 1 was bordered by a row of taller trees 6 m to the east. Different shades indicate different treatments: white = no trees, grey = low tree density, black = high tree density.
