# Supplementary material for: How Does Tree Density Affect Water Loss of Peatlands? A Mesocosm Experiment
Source: PLoS One. 2014 Mar 14;9(3):e91748. doi: 10.1371/journal.pone.0091748 (PMC3954773; doi:10.1371/journal.pone.0091748)
Supplement: Figure S2 — Tree density effects on subsurface temperature. (DOCX) [file pone.0091748.s002.docx]

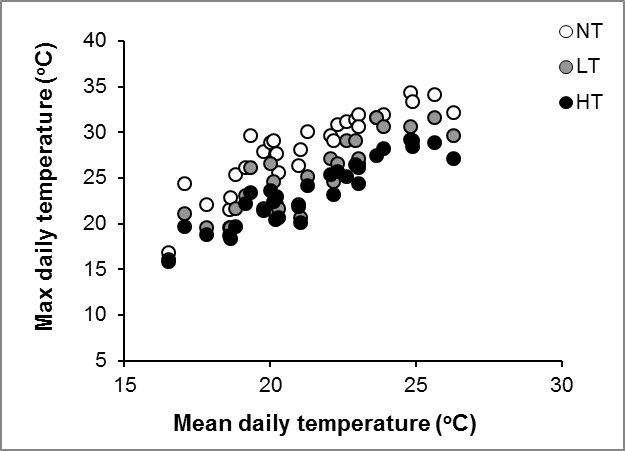


**Figure S2.** **Tree density effects on subsurface temperature**. Relationship between the mean daily temperature in control mesocosms and the maximum daily temperatures for all mesocosms in July 2010. Measurements were taken 1 cm below the moss surface in the middle of 1 (low density) or 2 (control and high density) mesocosms of the same block. Standard errors between replicates remained below 5%. Grey tones indicate different tree densities.
