# Supplementary material for: How Does Tree Density Affect Water Loss of Peatlands? A Mesocosm Experiment
Source: PLoS One. 2014 Mar 14;9(3):e91748. doi: 10.1371/journal.pone.0091748 (PMC3954773; doi:10.1371/journal.pone.0091748)
Supplement: Table S1 — Water table fluctuations under natural field conditions. (DOCX) [file pone.0091748.s003.docx]

**Table S1. Water table draw down and characteristics of birch stands in a natural bog.**

| **Plot** | **Area** | **Birch density** | **Sum basal area** | **LAI** | **Water table summer 2008** |
| --- | --- | --- | --- | --- | --- |
|  | *m^2^* | *m^-2^* | *m^2^* |  | *cm below moss surface* |
| 1 | 106 | 0.7 | 0.024 | 0.5 | 31 |
| 2 | 98 | 1.0 | 0.054 | 1.0 | 33 |
| 3 | 107 | 1.0 | 0.043 | 0.8 | 33 |
| 4 | 106 | 1.1 | 0.016 | 0.5 | 11 |
| 5 | 91 | 1.7 | 0.017 | 0.3 | 19 |
| 6 | 101 | 1.7 | 0.030 | 0.5 | 26 |
| 7 | 125 | 0.9 | 0.008 | 0.2 | 26 |
| 8 | 122 | 0.9 | 0.016 | 0.4 | 20 |
| 9 | 102 | 0.3 | 0.005 | - | 11 |
| 10 | 114 | 1.1 | 0.030 | 0.7 | 25 |
| 11 | 97 | 3.6 | 0.056 | 0.9 | 24 |
| 12 | 124 | 2.0 | 0.040 | 0.9 | 21 |
| 13 | 104 | 0.2 | 0.003 | - | 3 |
| 14 | 123 | 2.2 | 0.087 | 1.2 | 29 |

In winter 2008-2009 14 plots were set out in the Haaksbergerveen bog reserve (52°7'N, 6°46'E). The plots were representative for the range in birch densities observed in this bog reserve. The area of the plots was assessed using DGPS. All trees within this area were counted and their circumference was measured at the base of the stem, just above the moss surface. In the middle of each plot a piezometer was placed. Water tables were measured at monthly intervals over the summer of 2008. Area represents the area of each field plot. Birch density indicates the number of trees and tree saplings per unit of area. Sum of basal area is the sum of all birch-stem areas within each plot. Water table indicates the deepest water table measured for the summer in 2008 (end of July). Plots with floating peat layers (4, 9 and 13) have been indicated in grey. The other plots were situated on solid peat.
