# Supplementary material for: How Does Tree Density Affect Water Loss of Peatlands? A Mesocosm Experiment
Source: PLoS One. 2014 Mar 14;9(3):e91748. doi: 10.1371/journal.pone.0091748 (PMC3954773; doi:10.1371/journal.pone.0091748)
Supplement: Table S2 — Storage coefficients (S) of mesocosms: values and measurement procedure. (DOCX) [file pone.0091748.s004.docx]

**Table S2. Storage coefficients per treatment per year.**

| **No Tree** | | **Low Tree** | | **High Tree** | |
| --- | --- | --- | --- | --- | --- |
| *2008* | *2010* | *2008* | *2010* | *2008* | *2010* |
| 0.25 | 0.32 | 0.29 | 0.30 | 0.35 | 0.37 |
| 0.24 | 0.35 | 0.28 | 0.30 | 0.23 | 0.32 |
| 0.25 | 0.33 | 0.25 | 0.32 | 0.27 | 0.35 |
| 0.27 | 0.36 | 0.20 | 0.53 | 0.28 | 0.33 |
| 0.24 | 0.35 | 0.23 | 0.34 | 0.28 | 0.35 |

Values represent mesocosm specific storage coefficients measured over the upper 30-40cm of peat. Storage coefficients were significantly higher in 2010 than in 2008, but remained

unaffected by tree density. (RANOVA-_Fε1.0,df1_ = 81, P<0.001). Numbers in grey indicate statistical outliers.

**Measurement procedure**

The storage coefficient, S, is the volume of water per unit of surface area per unit of water table change. The storage coefficient of the upper 30-40cm of peat was measured for each mesocosm both in the first (2008) and final year (2010) of the experiment by alternately pumping out water of the mesocosms and measuring the resulting change in water table. The relationship between storage and water table was best explained by a linear relationship, indicating the coefficient remained constant with depth. R^2^ of the mesocosm-specific linear regressions ranged between 0.68 and 0.99 in 2008 (mean = 0.88 ± 0.09 SD) and between 0.94 and 1.0 in 2010 (mean =0.99 ± 0.09 SD). The 2008 and 2010 values were averaged, yielding mesocosm –specific values of S that were used to calculate mesocosm -specific changes in water storage (ΔW, see methods MS).

S was determined early April 2008 and 2010, when water tables were closest to the surface. Water table level was measured within the vertical drainage pipe of each mesocosm relative to a fixed point. In 2008, we pumped a total of 40 L out of each mesocosm in steps of 5 litres. Between each step, we let the water table stabilise for at least 4 hours before again measuring the water table and pumping out the next 5 L. In 2010, we pumped out 60 L in steps of 20 litres, allowing 24 hours for stabilisation. Within one week after each measurement of S, heavy rainfall restored the water tables in the mesocosms.
