# Supplementary material for: How Does Tree Density Affect Water Loss of Peatlands? A Mesocosm Experiment
Source: PLoS One. 2014 Mar 14;9(3):e91748. doi: 10.1371/journal.pone.0091748 (PMC3954773; doi:10.1371/journal.pone.0091748)
Supplement: Table S3 — Leaf area index (LAI) of plots and mesocosms. (DOCX) [file pone.0091748.s005.docx]

**Table S3. Leaf area index (LAI) of plots and mesocosms.**

|  | **Year** | **Low Tree** | | **High Tree** | | **Statistics** | |
| --- | --- | --- | --- | --- | --- | --- | --- |
|  |  | *mean* | *SE* | *mean* | *SE* | *Treament* | *Block* |
| LAI (plots) | 2008 | 0.3a | 0.01 | 1.2b | 0.08 | F_df1_ = 143*** | ns |
|  | 2009 | 0.5a | 0.04 | 2.4b | 0.13 | F_df1_ = 218*** | ns |
|  | 2010 | 1.5a | 0.22 | 3.3b | 0.13 | F_df1_ = 26** | ns |
| LAI (mesocosms) | 2009 | 0.5a | 0.03 | 1.2b | 0.10 | F_df1_ = 41** | ns |
|  | 2010 | 0.9a | 0.05 | 1.8b | 0.19 | F_df1_ = 42** | ns |

Values represent mean and standard error (n = 5) for the experimental years. Plot-LAI in 2009 and 2010 was measured with an LAI 2000, plot-LAI in 2008 and mesocosm-LAI were derived from the total stem area of the birches (see below). Different letters denote statistically significant differences between birch density treatments for the same year (row-wise comparisons). Data were analysed for each year separately, using 2-way ANOVAs with treatment as factor and block as random factor. Ns = *P* < 0.10, ** = 0.05 > *P* ≤ 0.01, *** = *P* < 0.01.

**A**

**Plot-LAI in 2008 and mesocosm-LAI**

Plot-LAI in 2008 and mesocosm-LAI were derived from the stem area of the trees, applying an empiric relationship between LAI and stem area: LAI = 1447 $\times$ (stem area/plot area) + 0.19 (R^2^ = 0.90). This empiric relationship was derived from measurements on natural birch stands in the Haaksbergerveen bog reserve.

In the Haaksbergerveen bog reserve we derived a relationship between total stem area and LAI in 2009. Here we selected 12 plots of 10 x 10m differing in birch density. The stem area of all birches was measured just above the moss surface. The same year we measured LAI within the plots using an LAI-2000 (LI-COR) on an overcast day. Plot LAI was based on 12 measurements under the birch canopy and 4 outside the birch canopy at each plot corner. The measurements were taken along a cross through the centre of each plot at about 50 cm above the surface. With the plots we covered a range of LAI = 0.2 to LAI = 1.2 (see Table S1). The total area of the birch stems was divided by the plot area and related to LAI. A linear relationship (R^2^ = 0.90) gave a better fit than a non-linear relationship (R^2^ = 0.86).

In Wageningen we measured the stem area of all the birches in the plots, both inside (above moss surface) and outside (about 15 cm above surface) the mesocosms in 2008. In 2009 and 2010 we only measured stem area of the birches inside the mesocosms.
